# Diagnosing Coronavirus Disease 2019 (COVID-19): Efficient Harris Hawks-Inspired Fuzzy K-Nearest Neighbor Prediction Methods

**DOI:** 10.1109/ACCESS.2021.3052835

**Published:** 2021-01-19

**Authors:** Hua Ye, Peiliang Wu, Tianru Zhu, Zhongxiang Xiao, Xie Zhang, Long Zheng, Rongwei Zheng, Yangjie Sun, Weilong Zhou, Qinlei Fu, Xinxin Ye, Ali Chen, Shuang Zheng, Ali Asghar Heidari, Mingjing Wang, Jiandong Zhu, Huiling Chen, Jifa Li

**Affiliations:** 1 Department of Pulmonary and Critical Care MedicineAffiliated Yueqing Hospital, Wenzhou Medical University 26453 Yueqing 325600 China; 2 Department of Pulmonary and Critical Care MedicineThe 1st Affiliated Hospital, Wenzhou Medical University 26453 Wenzhou 325000 China; 3 The Second Clinical CollegeWenzhou Medical University 26453 Wenzhou 325000 China; 4 Department of PharmacyAffiliated Yueqing Hospital, Wenzhou Medical University 26453 Yueqing 325600 China; 5 Department of UrologyAffiliated Yueqing Hospital, Wenzhou Medical University 26453 Yueqing 325600 China; 6 School of Surveying and Geospatial Engineering, College of EngineeringUniversity of Tehran 48425 Tehran 1417466191 Iran; 7 Department of Computer ScienceSchool of ComputingNational University of Singapore 37580 Singapore 117417; 8 Institute of Research and Development, Duy Tan University 374802 Da Nang 550000 Vietnam; 9 Department of Surgical OncologyAffiliated Yueqing Hospital, Wenzhou Medical University 26453 Yueqing 325600 China; 10 College of Computer Science and Artificial IntelligenceWenzhou University 26495 Wenzhou 325035 China

**Keywords:** COVID-19, coronavirus, fuzzy K-nearest neighbor, Harris hawk optimization, disease diagnosis, feature selection

## Abstract

This study is devoted to proposing a useful intelligent prediction model to distinguish the severity of COVID-19, to provide a more fair and reasonable reference for assisting clinical diagnostic decision-making. Based on patients’ necessary information, pre-existing diseases, symptoms, immune indexes, and complications, this article proposes a prediction model using the Harris hawks optimization (HHO) to optimize the Fuzzy K-nearest neighbor (FKNN), which is called HHO-FKNN. This model is utilized to distinguish the severity of COVID-19. In HHO-FKNN, the purpose of introducing HHO is to optimize the FKNN’s optimal parameters and feature subsets simultaneously. Also, based on actual COVID-19 data, we conducted a comparative experiment between HHO-FKNN and several well-known machine learning algorithms, which result shows that not only the proposed HHO-FKNN can obtain better classification performance and higher stability on the four indexes but also screen out the key features that distinguish severe COVID-19 from mild COVID-19. Therefore, we can conclude that the proposed HHO-FKNN model is expected to become a useful tool for COVID-19 prediction.

## I. Introduction

Coronavirus disease 2019 (COVID-19) is a highly contagious viral disease, and the World Health Organization (WHO) declared that the COVID-19 was an international public health emergency [1], [2]. First described COVID-19 in December 2019 in Wuhan, Hubei Province, China. The ongoing outbreak of COVID-19 is affecting multiple countries in the world [1]. Until Mar 11th, 2020, 118,326 cases of COVID-19 were diagnosed worldwide, including 80,955 cases in China and 37,371 cases outside China. Additionally, 4,292 deaths have been triggered by COVID-19 [3]. Many countries are facing increased pressures on health care resources. Up to now, a great deal of studies is focused on using traditional statistical methods to identify risk factors of COVID-19 patients. As an example, older age, pre-existing diseases, abnormal liver function, and T-lymphocyte count were correlated closely with COVID-19 progression and prognosis [4]–[6]. However, traditional statistical methods could not rapidly identify changes in COVID-19 patient’s status during the outbreak. Therefore, there is a crucial need to progress a useful forecasting tool for COVID-19 and quickly categorize illness severity.

Currently, the importance of computational, mathematical, and surveillance-based methods for the study of infectious diseases continues to increase [7]. Machine learning-based methods are increasingly being used to diagnose disease, develop prediction models, and identify risk factors [8]. The benefits of machine learning include improving health professionals’ ability to establish diagnosis or prognosis; it will replace much of the work of radiologists and anatomic/clinical pathologists; it will improve the accuracy of diagnosis [9]. Therefore, machine learning is an indispensable tool for clinicians seeking to understand patient-individualized treatment better. We use a machine learning strategy to identify COVID-19 patients at high risk for severe illness and prioritize their hospitalization. It may contribute to reduce patient mortality and reduce the burden on healthcare resources.

Optimization depends on the form of the problem we deal with. It is almost possible to reach any form according to the decision-makers preferences. These problems can be modeled as many-objective [10], [11], memetic [12], robust [13], multiobjective [14], large scale [15], [16], fuzzy [17], and single-objective optimization. These forms and the growing demand for their solvers have raised many challenges in data science. Core problems demanding optimization are not limited to healthcare systems, but technologies such as the neural networks [18], water-energy optimization [19], image boost optimization [20], decision-making systems [21]–[23], temperature optimization [24], deployment optimization in sensor networks [25], sustainable development [26]–[28], parameter optimization [29], optimal resource allocation [30], deep learning tasks [31]–[33], mechanical parameters optimization [34], and many other potentials and connected domains [35]–[39]. One of the main classes are solvers with an evolutionary basis. This optimization algorithm works based on an evolving swarm with stochastic updating rules. They have found a good application effect in many scenarios [40]–[45]. At present, as a new single-objective algorithm, Harris Hawks Optimizer (HHO)^1^ has been widely concerned like other optimizers and their applications such as Particle Swarm Optimizer (PSO) [46], [47], Whale Optimizer (WOA) [48], Differential Search (DS) [49], Differential Evolution (DE) [50], Slime Mould Algorithm (SMA)^2^ [51], Monarch Butterfly Optimization (MBO) [52], and Moth Search Algorithm (MSA) [53].^1^Download the codes at https://aliasgharheidari.com/HHO.html^2^Download the codes at https://aliasgharheidari.com/SMA.html

HHO not only has strong plasticity but also has been widely used in other fields. Many HHO variants have been proposed recently [54]–[57]. Elaziz *et al.* [58] proposed an improved Harris-Hawks Optimizer (HHO) to solve a multilevel image segmentation problem’s global optimization problem and determine the optimal threshold. A large number of results and comparisons show that SSA has a strong ability to improve HHO. Gupta *et al.* [59] put four effective strategies into the traditional HHO, such as putting forward a nonlinear prey energy parameter, different fast diving, greedy selection mechanism, and learning based on opposites. Experimental results show that the proposed m-HHO can be a useful optimization tool for solving global optimization problems. Shao, Shao *et al.* [60] proposed a new rolling bearing fault diagnosis method based on variational mode decomposition (VMD), time-shifting multiscale discrete entropy (TSMDE), and support vector machine (SVM) optimized by vibration Harris Hawks Optimization algorithm. The outcomes show that this routine has better diagnostic performance than other comparison methods. Tikhamarine *et al.* [61] Combined multi-layer perceptron (MLP) neural network and least squares support vector machine (LSSVM) data-driven technology with advanced natural heuristic optimizer (HHO) to simulate rainfall-runoff relationship. The experimental results show that the mixture of HHO and LSSVM can obtain high accuracy of runoff prediction.

Machine learning is widely used in the medical field. For example, Abbasi *et al.* [62] proposed a new method for solving large-scale stochastic operation optimization problems (SOPs) using a machine learning model and applying the proposed decision-making method of blood unit transportation in the hospital network. The results show that compared with the current strategy, the average daily cost is reduced by 29% with the trained neural network model. In comparison, the average daily cost can be reduced by 37% with the proper optimal strategy. Amiri *et al.* [63] used photonic crystal structure and machine learning technology to calculate the concentration of potassium chloride, urea, and glucose (PUG) in human blood to achieve accurate measurement. Moreover, at this article’s finale, a mathematical model is revealed to obtain the output power and potassium chloride changes, urea, and glucose concentrations. Ayyıldız and Arslan Tuncer [64] used red blood cell index and machine learning technology, including support vector machine (SVM) and k-nearest neighbor (KNN), to differentiate IDA from Î - thalassemia. Instead, it employs the neighborhood component analysis feature selection (NCA) technique to select the dataset’s features. Their obtained results point to that the RBC indices can result in higher efficacy than those described in the other works. Banerjee *et al.* [65] used machine learning (ML), artificial neural network (ANN) [18], and a humble statistical test to recognize sars-cov-2 positive patients. This new method can significantly improve initial screening for patients with limited PCR based diagnostic tools. Rammurthy and Mahesh [66] designed the wHHO by combining whale optimization algorithm (WOA) with HHO. They applied it to the tumor automatic classification model. Experiments show that the method of deep CNN based on WHHO is better than other methods.

This study aims to develop efficient frameworks using the Harris hawk’s optimizer (HHO), which trains a fuzzy k-nearest neighbor (FKNN) model. Then, the optimized HHO-FKNN is substantiated for the first time to diagnose the severity of COVID-19. The active model is built using the info about patients’ necessary information, pre-existing diseases, symptoms, immune indexes, and complications. In the developed method (HHO-FKNN), HHO was employed to train an FKNN model and to explore the critical risk factors of COVID-19 infected people at the same time. In the experiment, HHO-FKNN is compared with the other machine learning methods like grey wolf optimizer (GWO)-based FKNN (GWO-FKNN), support vector machines (SVM), and random forest (RF). It is shown that the established HHO-FKNN method performs much better than its peers in terms of four evaluation metrics, including the classification accuracy (ACC), sensitivity, specificity, and Matthews Correlation Coefficients (MCC). Hence, the main contributions of this study can be listed as follows:

(a) The well-established HHO was successfully applied to tackle the parameter tuning and feature selection for FKNN.
(b) The proposed HHO-FKNN is employed for the diagnosis of the severity of COVID-19 for the first time.
(c) It is the first time to diagnose the severity of COVID-19 based on an immune index.
(d) The established HHO-FKNN model outperforms other evolutionary-based competitors.

The structure of this article is as bellow. Section 2 described the data and proposed the HHO-FKNN model in detail. The experimental arrangement and marks are explained in Section 3. Section 4 presents the discussions. The conclusions and upcoming works are presented in Section 5.

## II. HHO-FKNN Method

**FIGURE 1.**
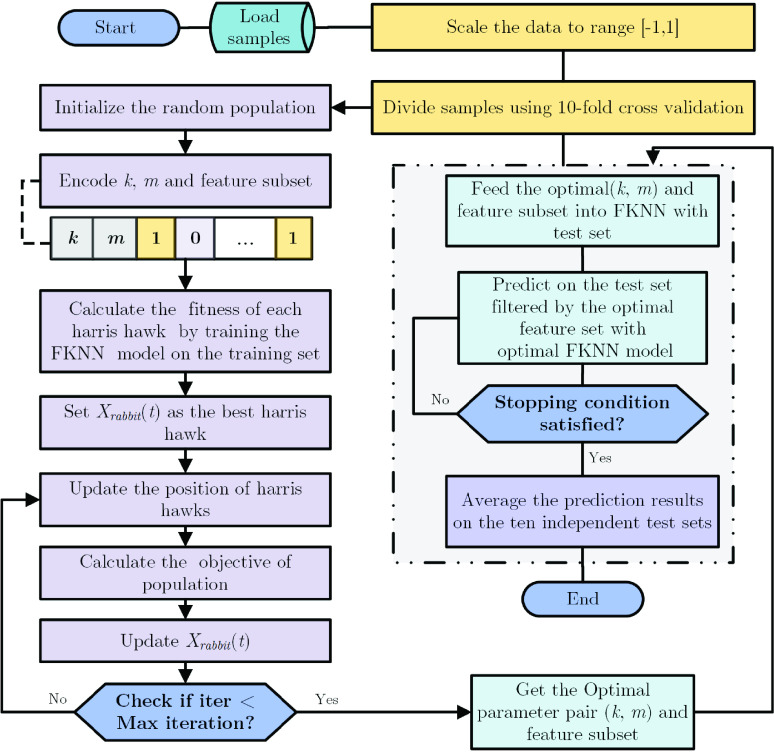
Flowchart of HHO-FKNN.

The flowchart of HHO-FKNN is exposed in Figure 1. The whole procedure comprises data acquisition, standardization, feature selection, and classification. The first phase is to normalize the records, then using the HHO approach to select the informative features from the data samples and optimize the two critical parameters of the FKNN model. Then the FKNN classifier is trained again by using the optimal parameters and feature subset. Finally, the optimal FKNN classifier is taken to determine whether the specific patient is severe or non-severe. The commonly used 10-fold cross-validation (CV) scheme is used to divide the data and obtain more accurate and unbiased experimental results, often adopted by many studies [67]–[74].

### A. Parameter Optimization and Feature Selection by HHO

Healthcare and diagnosis systems have many hardware and software aspects considered and modeled [75], [76]. Any inaccuracy in the diagnosis can lead to a crisis [77]. This research focuses on developing a hybrid HHO-FKNN prediction core to be utilized for diagnostic purposes. In this model, the feature selection core utilizes a binary model of HHO. As we know, the primary method of HHO is a continuous approach verified on a set of problems with no binary variable. HHO was invented by Heidari *et al.* [78] as a new swarm-based algorithm for solving a set of unconstrained or even constrained cases. This method also has its exclusive features based on hawks and rabbits’ greedy actions in wildlife [79]. This method found its widespread applications in dealing with different problems [80]–[82].

This research deals with a feature selection problem that inherits both continuous and binary optimization aspects. From the other side, we know the initial HHO is not compatible with binary spaces. However, we need to optimize both types of variables. Thus, we aim to advance the hybrid HHO by combining the continuous variant and the binary type to optimize any parameters.

### B. Classification Based on FKNN

FKNN is used as a core prediction engine, which is used to perform classification tasks after obtaining the optimal parameters and feature subset. FKNN [83], [84] classifier is developed based on the traditional k-nearest neighbor (KNN) classifier and has been widely considered from the time when it was initially suggested [85], [86], [86]–[88]. Compared with procedures such as extreme learning machines [89]–[94], deep learning methods [95]–[97], and support vector machines [70], [17], [98]–[103], FKNN is much simpler and can return outcomes that can be more easily understood.

Up to now, FKNN presented its capacity as a unique aspect of neighbor classification and case-based learning [104]. It is one of the most significant characteristics of FKNN to represent inaccurate information and provide samples belonging to related categories. After being allocated a membership, we can classify each sample into a class with the highest membership value. Many works utilized FKNN because of its competitive advantages. For examples, it found its application in various scenarios, including slope collapse prediction scenarios [105], protein identification and prediction scenarios [106], [107], medical diagnosis cases [87], [108], bankruptcy prediction models [86], and grouting activity prediction scenarios [109]. Logically, there is no perfect model and method in the artificial intelligence area. For instance, FKNN suffers from some issues; one of them is its dependency on two key parameters, the fuzzy intensity factor ($m)$ and the number of neighbors ($k)$. Therefore, these two limits have to be adjusted correctly to attain a superior classification efficacy. In this research, we employed the HHO to adjust the parameters of FKNN.

To implement the fuzzy $k$-NN system, we follow the next operations:

Step 1: Consider
  \begin{equation*} X_{R} =\{ x_{i} \}_{i=1}^{m_{R}}\end{equation*}
  to be the reference set we have at hand and
  \begin{equation*} W=\{ w_{i} \}_{i=1}^{m_{R}}\end{equation*}
  be a set of $i$-dimensional vectors.
Step 2: \begin{equation*}w_{i} =(w_{i,1},w_{i,2},\ldots ,w_{i,l} )\quad \sum \limits _{j=1}^{l} {w_{i,j} =1 \textrm {and} 0\le w_{i,j} \le 1}\end{equation*}
  For every $w_{i,j} $, $1\le i\le m_{R} $, $1\le j\le l$, and $l$ denotes the number of classes.
Step 3: For each $x$ that we want to classify, we can attain the set $K$ of indices that correspond with the “$k$ nearest neighbors of $x$ in $X_{R}$ and the fuzzy decision-vector” [83].
  \begin{equation*} v={\left ({ {\sum \limits _{x\in K} {w_{s}}} }\right )} /k\end{equation*}
Step 4: If the user is involved in a non-fuzzy decision, ties are wrecked randomly or by the single NN law. If all $w_{i,j}$, $1\le i\le m_{R} $, $1\le j\le l$, are equivalent to 0 or 1, then we can consider the fuzzy $k$-NN instruction equivalents to the normal $k$-NN rule.

### C. Proposed HHO-FKNN

To utilize and explore the potential of KNN, its parameters are optimized and adjusted using the HHO. It is also appointed to determine the optimal feature subsets in the datasets. Here, we describe the main steps of feature selection and parameter optimization of the proposed HHO-FKNN model:

Step 1: Load samples and scale the data.
Step 2: Divide samples using 10-fold cross-validation.
Step 3: Initialize the input parameters of HHO. These parameters are population, population size, bounds, space dimension, and numbers of iterations.
Step 4: Encode $k$, *m,* and feature subset.
Step 5: Cross-boundary treatment and calculation of population fitness.
Step 6: Update parameters E.
  \begin{equation*} E=2E_{0} \left ({ {1-\frac {t}{T}} }\right )\tag{1}\end{equation*}
  where $E_{0} $ is a random number of [−1, 1].
Step 7: Attain the fitness value with (*k,* $m)$ and the chosen features for each swarm member referring to the following rule.
  \begin{align*} \begin{cases} \displaystyle f_{1} =\frac {\sum \limits _{i=1}^{K} {acc_{i}}}{K} \\ \displaystyle f_{2} =1-\frac {\sum \limits _{j=1}^{n} {bin_{j}}}{n} \\ \displaystyle \\ \displaystyle f=\alpha \times f_{1} +\beta \times f_{2} \end{cases}\tag{2}\end{align*}
  Here we determine the objective function needs to be minimized. The first sub-objective function $f_{1}$ denotes the average accuracy degree realized by the FKNN through K-fold CV, where K = 5 and acci denote the ith fold CV’s accuracy. For another sub-objective rule (function) $f_{2}$, $bin_{j}$ shows the jth feature’s binary value, and $n$ denotes the full number of features. In the resulted objective formulation, which is shown using $f$, we have two scaling factors. One of them is $\alpha $, which shows the weight of the accuracy term decided by the user. Simultaneously, the other, denoted by $\beta $, indicates the scale weighting for the selected features [110].
Step 8: Choose the first best hawk (solution) with extreme fitness value and keep them as $X_{rabbit}(t)$.
Step 9: Update the location according to the three main parts of “soft besiege,” “hard besiege,” and “soft besiege with rapid progressive divides.” The updating rule that hawks follow to catch the rabbits during the “soft besiege” phase is as follow:
  \begin{align*} X\left ({ {t+1} }\right )=&\Delta X(t)-E\left |{ {JX_{rabbit} (t)-X(t)} }\right |\qquad \tag{3}\\ \Delta X(t)=&X_{rabbit} (t)-X(t)\tag{4}\end{align*}
  where $J$ is a random number of [0, 2.
  The updating equation of the “hard besiege” step is expressed as follows:
  \begin{equation*} X\left ({ {t+1} }\right )=X_{rabbit} (t)-E\left |{ {\Delta X(t)} }\right |\tag{5}\end{equation*}
  The updated formula of “soft besiege with progressive rapid divides” is as follow:
  \begin{align*} Y=&X_{rabbit} (t)-E\left |{ {JX_{rabbit} (t)-X(t)} }\right | \tag{6}\\ Z=&Y+S\times LF(D)\tag{7}\end{align*}
  D is the case’s dimension we want to solve, and $S$ indicates a random vector by size $1\times D$. *LF* is the call function of Levy’s fight.
Step 10: Calculate the objective of the population.
Step 11: Go to step 3 if the maximum number of iterations has not been reached.
Step 12: Get the optimal parameter pair ($k$, $m)$ and feature subset and Feed the optimal ($k$, $m)$ and feature subset into FKNN with the test set.
Step 13: Go to step 12 if the condition is not satisfied.
Step 14: Average the prediction results on the ten independent test sets.
Step 15: Print and post-process the first two elements of $X_{rabbit}(t)$ as the optimal FKNN pair (*k,* $m)$ and the other $n$ dimensions of the binary values of $X_{rabbit}(t)$ as the indicators of the finest subset of the feature.

## III. Experimental Setup and Results

This section compares the proposed CPA with several conventional and recent optimizers in the field. All experiments were conducted on Windows Server 2008 R2 operating system with Intel (R) Xeon (R) Silver 4110 CPU (2.10 GHz) (2.10GHz) and 128 GB of RAM. We coded all algorithms for comparison on the MATLAB R2014b.

### A. Data Collection

A retrospective review of medical records was conducted for 47 patients with COVID-19 pneumonia admitted to the *Affiliated Yueqing Hospital of Wenzhou Medical University* (Yueqing, China) from Jan 21 to Mar 10, 2020. Severe acute respiratory syndrome coronavirus 2 (SARS-CoV-2) testing performed on admission on all patients with COVID-19 pneumonia using real-time reverse transcriptional polymerase chain reaction (RT-PCR). All patients were SARS-CoV-2-positive. The COVID-19 pneumonia was spotted referring to the New Coronavirus Pneumonia Prevention and Control Program published by the National Health Commission of the People’s Republic of China in 2020 [111]. To simplify the data analysis, patients were categorized into two groups, severe ($n = 21$) and non-severe cases ($n = 26$). Patients were required to encounter at least one of the next criteria for the diagnosis of severe COVID-19:

(i) Respiratory distress with respiratory frequency ≥ 30/min;
(ii) Resting oxygen saturation ≤ 93%;
(iii) Oxygenation index (arterial oxygen pressure (PaO_2_, mmHg)/fraction of inspired oxygen (FiO_2_) ratios, PaO_2_/FiO_2_) ≤ 300 mmHg.

A detailed description of the utilized database for research is tabulated in Table 1. This study was permitted by the Ethics Committees of the *Affiliated Yueqing Hospital of Wenzhou Medical University* (code: 202000002) and complied with the Helsinki declaration. The clinical parameter and immunological indices were analyzed by an independent sample t-test, using SPSS version 21.0 (IBM, Somers, NY, USA). A p-value < 0.05 was considered to be statistically significant. Detailed results of the statistical analysis are described in Table 2.

**TABLE 1:** Description of 31attributes

No.	Feature	Detailed description
F1	Gender	Male = 0; Female = 1
F2	Age	Severe cases (mean, standard deviation) = 61.43±17.64 Non-severe cases (mean, standard deviation) = 42.58±11.62
F3	CD4 T-lymphocyte count (CD4 T)	Severe cases (mean, standard deviation) = 313.67±164.53 Non-severe cases (mean, standard deviation) = 719.00±238.63
F4	CD8 T-lymphocyte count (CD8 T)	Severe cases (mean, standard deviation) = 184.09±118.34 Non-severe cases (mean, standard deviation) = 464.36±143.39
F5	Contact history (CH)	Wuhan Sojourn = 1; Hubei Sojourn = 2; Wuhan patient contact = 3; Hubei patient contact = 4; Other regions patient contact = 5; No contact = 6
F6	Smoking history (SH)	No = 0; Yes = 1
F7	Drinking history (DH)	No = 0; Yes = 1
F8	Hypertension (HP)	No = 0; Yes = 1
F9	Diabetes mellitus (DM)	No = 0; Yes = 1
F10	Chronic kidney disease (CKD)	No = 0; Yes = 1
F11	Chronic liver disease (CLD)	No = 0; Yes = 1
F12	Chronic heart disease (CHD)	No = 0; Yes = 1
F13	Chronic lung disease (CLGD)	No = 0; Yes = 1
F14	Malignant tumor (MT)	No = 0; Yes = 1
F15	Fever	No = 0; Yes = 1
F16	Cough	No = 0; Yes = 1
F17	Sputum	No = 0; Yes = 1
F18	Nasal obstruction (NO)	No = 0; Yes = 1
F19	Sore throat (ST)	No = 0; Yes = 1
F20	Fatigue	No = 0; Yes = 1
F21	Myalgia	No = 0; Yes = 1
F22	Headache	No = 0; Yes = 1
F23	Nausea/Vomiting (N/V)	No = 0; Yes = 1
F24	Abdominal Pain (AP)	No = 0; Yes = 1
F25	Diarrhea	No = 0; Yes = 1
F26	Shock	No = 0; Yes = 1
F27	Vasopressor drug (VD)	No = 0; Yes = 1
F28	Acute kidney injury (AKI)	No = 0; Yes = 1
F29	Acute myocardial injury (AMI)	No = 0; Yes = 1
F30	Acute liver injury (ALI)	No = 0; Yes = 1
F31	Secondary infection (SI)	No = 0; Yes = 1

**TABLE 2:** Immunological & Clinical Parameter in Severe Patients and Non-Severe Patients

Index	Severe ($n =21$)	Non-severe ($n =26$)	p-value
Age (years)	61.43±17.64	42.58±11.62	0.000
CD4 T ($\mu $l)	313.67±164.53	719.00±238.63	0.000
CD8 T ($\mu $l)	184.09±118.34	464.36±143.39	0.000

Table 3 records the time relative values of one iteration of HHO-FKNN and GWO-FKNN. In the table, the relative value of HHO-FKNN running time is set as 1, and 1.07 is the running time of GWO-FKNN relative to HHO-FKNN. The larger the value is, the longer the running time.

**TABLE 3:** Comparison of CPU Running Time Between HHO-FKNN and GWO-FKNN

Algorithm	Relative running time	Ranking
HHO-FKNN	1	1
GWO-FKNN	1.07	2

### B. Experimental Setup

In this section, we need to run a set of experiments to substantiate the HHO-based model’s efficacy in diagnosing the COVID-19. The investigated methods, comprising HHO-FKNN and GWO-FKNN [112], were realized from scratch based on the software of MATLAB. We normalized the input data to be inside [−1, 1] in advance of the classification’s performance. The stratified 10-fold CV was employed with our tests to weigh the value of the classification results (costs) and guarantee not having biased results. The maximum iterations and the number of members in the swarm were set at 50 and 20, respectively. The searching variety for the two strictures in FKNN is determined as follows

The involved methods, HHO-FKNN and GWO-FKNN [112] were both implemented from scratch in the MATLAB environment. We have respected fair comparisons referring to the neural network literature [113]–[115]. There is no biased test due to advantageous testing conditions [116]–[120]. Before we perform the classification, we normalize the data into the range [−1, 1]. Also, we employed a 10-fold cross-validation CV for evaluating the efficacies to ensure unbiased results. There should be a limit for the time of execution, which is set to 50 iterations. Also, we require that each method starts with 20 agents inside the feature space. For searching ranges of dual factors in FKNN, we have $k~\in $ [1, 5], $m~\in $ [1, 5]. LIBSVM and RF tools^3^ were used to run SVM and RF models.^3^https://www.csie.ntu.edu.tw/~cjlin/libsvm/, and https://code.google.com/archive/p/randomforest-matlab.

### C. Experimental Results

To assess the HHO-FKNN with the feature selection (FS) method, we utilized well-regarded criteria such as classification accuracy (ACC), sensitivity, specificity, and Matthews’s correlation coefficient (MCC). Table 4 shows the four evaluation indicators’ specific values, including classification accuracy, MCC, sensitivity, specificity, and shows the results of four evaluation indicators using the mean and variance. The means of the four evaluation indicators of the model are 94.00%, 0.8891, 90.00%, and 96.67%, and their variances are 0.0966, 0.1791, 0.2108, and 0.1054, respectively. Also, according to the experimental results, it is obvious that the HHO algorithm can automatically obtain the optimal parameters of FKNN and provides the optimal feature subset at the same time.

**TABLE 4:** Classification Performance of HHO-FKNN in Terms of ACC, MCC, Sensitivity, and Specificity

Fold	Optimal features	ACC	MCC	Sensitivity	Specificity
No.1	{F2, F3, F4, F5, F8, F11, F13, F14, F16, F19, F20, F24, F26, F27, F31}	1.0000	1.0000	1.0000	1.0000
No.2	{F2, F3, F4, F6, F8, F10, F11, F14, F15, F16, F17, F19, F20, F27, F30, F31}	0.8000	0.6124	0.5000	1.0000
No.3	{F2, F3, F4, F5, F6, F7, F8, F9, F13, F16, F22, F23, F28, F29, F30, F31}	1.0000	1.0000	1.0000	1.0000
No.4	{F2, F3, F4, F5, F6, F7, F8, F10, F14, F15, F20, F29, F30}	1.0000	1.0000	1.0000	1.0000
No.5	{F2, F3, F4, F5, F6, F8, F13, F17, F18, F20, F22, F24, F25, F27, F30}	1.0000	1.0000	1.0000	1.0000
No.6	{F2, F4, F8, F11, F14, F15, F18, F20, F23, F27, F30}	1.0000	1.0000	1.0000	1.0000
No.7	{F2, F3, F5, F6, F7, F8, F11, F12, F13, F14, F20, F25, F26, F28, F30}	0.8000	0.6124	0.5000	1.0000
No.8	{F2, F3, F4, F6, F7, F8, F9, F11, F13, F15, F16, F18, F20, F24, F26, F31}	1.0000	1.0000	1.0000	1.0000
No.9	{F2, F3, F8, F10, F12, F14, F21, F23, F26, F30}	1.0000	1.0000	1.0000	1.0000
No.10	{F2, F3, F4, F11, F14, F15, F16, F19, F25, F27, F28, F29, F30}	1.0000	1.0000	1.0000	1.0000
AVG.	–	0.9400	0.8891	0.9000	0.9667
STD	–	0.0966	0.1791	0.2108	0.1054

**FIGURE 2.**
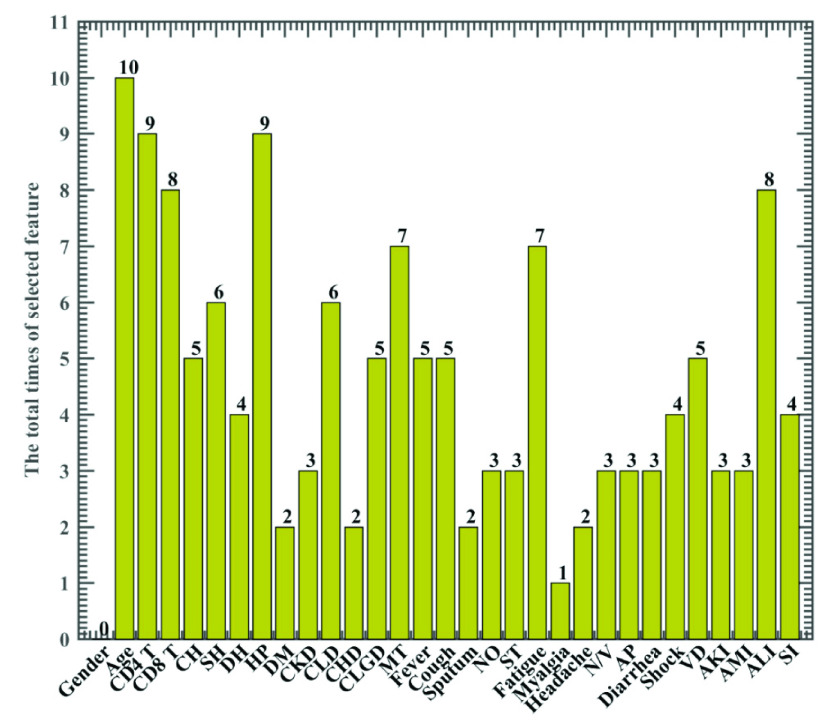
Frequency of the features obtained by HHO-FKNN via the 10-fold CV.

The frequency of each feature selected by HHO-FKNN via a 10-fold CV procedure is illustrated in Figure 2. As shown, As shown, Age, CD4 T, HP, CD8 T, ALI, and Fatigue are the five features with the highest frequency, and they seem 10, 9, 9, 8, 8, and 7 times, respectively. It means they contain the most discriminative information for recognizing the severe patients and non-severe patients. Therefore, these five informative factors need to be focused on clinically.

**FIGURE 3.**
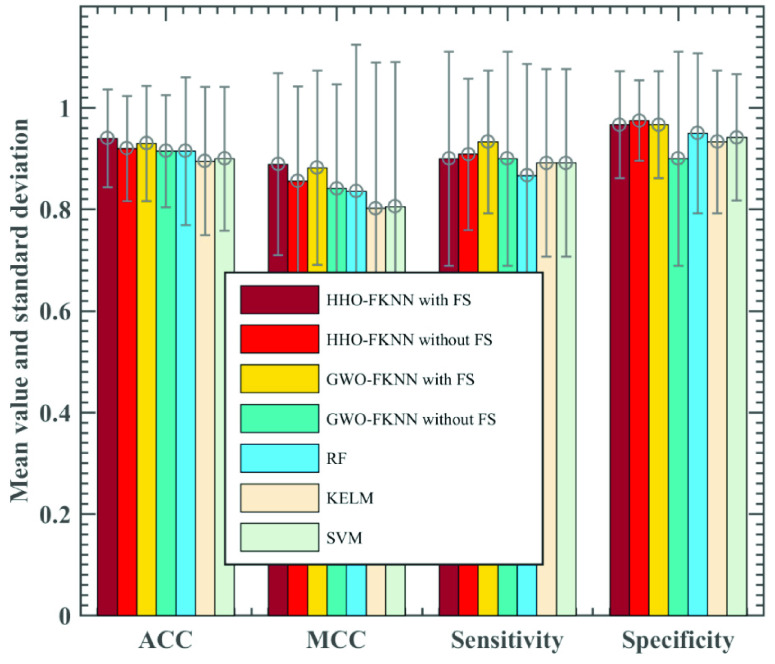
Classification efficacy of seven models based on the ACC, MCC, sensitivity, and specificity.

To validate the technique’s success, it is compared with six other operational machine learning models, including HHO-FKNN without FS, GWO-FKNN with FS, and GWO-FKNN without FS, RF, KELM, and SVM. According to the comparison results shown in Figure 3, the HHO-FKNN with the FS model is superior to the HHO-FKNN without the FS model in both the classification accuracy rate and the Matthews correlation coefficient. Its variance on these two indicators is also less than that of the HHO-FKNN without FS model. However, in terms of sensitivity and specificity, the HHO-FKNN with the FS model is inferior to the HHO-FKNN without the FS model. Its variance on these two indicators is also more significant than that of the HHO-FKNN without the FS model. Therefore, it is evident that the HHO-FKNN with the FS model with the feature selection algorithm has a better accuracy. The following are HHO-FKNN without FS, GWO-FKNN without FS, RF, SVM, and KELM. Besides, the accuracy of RF and GWO-FKNN without FS is the same HHO-FKNN with FS is two percentage points higher than HHO-FKNN without FS, and the variance of KELM is the largest, reaching 0.1462. In terms of the MCC evaluation index, HHO-FKNN with the FS model still achieved the best results, followed by GWO-FKNN with FS, which was 0.7 percentage points lower than HHO-FKNN with FS, HHO-FKNN without FS ranked third, which was 3.33 percentage points lower than HHO-FKNN with FS. Followed by GWO-FKNN without FS, RF, SVM, and KELM, KELM, and SVM are not much different; HHO-FKNN with FS has the smallest variance, and RF variance is the largest, reaching 0.2893. In terms of sensitivity evaluation index, GWO-FKNN with FS model has the best evaluation effect, followed HHO-FKNN without FS model has only 2.5 percentage points difference with it, HHO-FKNN with FS and GWO-FKNN without FS is the same, HHO-FKNN with FS model is 0.83 percentage points lower than HHO-FKNN without FS model. Finally, the values of KELM and SVM are the same, the variance of GWO-FKNN with FS is the lowest, and the variance of RF is the largest, reaching 0.2194. In terms of specific evaluation indexes, HHO-FKNN without the FS model has the best results and the smallest variance, followed by GWO-FKNN with FS and HHO-FKNN with FS are the same. They are 0.83 percentage points lower than HHO-FKNN without FS. The next models are RF, SVM, and KELM. The worst is GWO-FKNN without FS, which is only 90.00%. GWO-FKNN without FS has the most considerable variance, reaching 0.2108.

**FIGURE 4.**
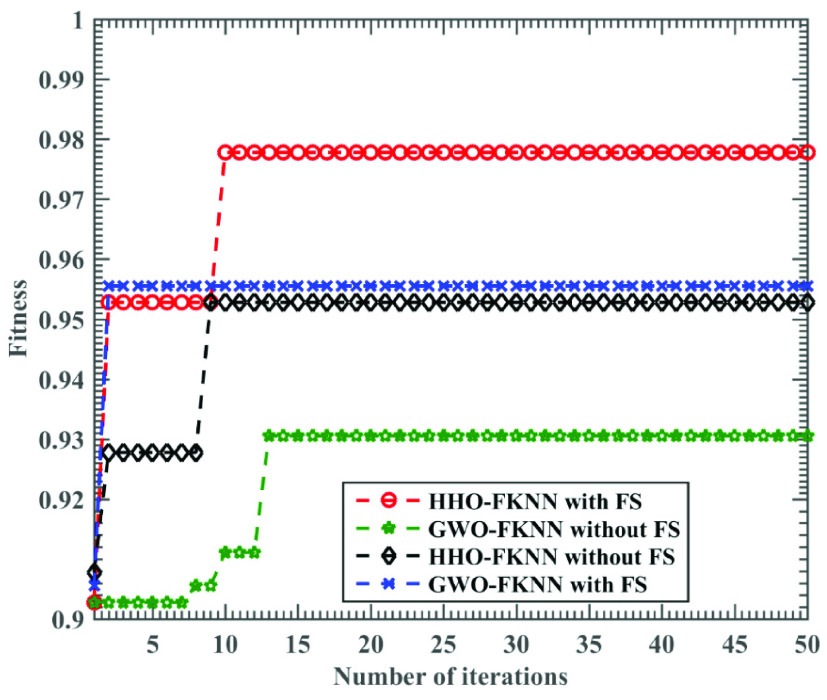
Relationship between training accuracy of HHO-FKNN with FS, HHO-FKNN without FS, GWO-FKNN with FS, and GWO-FKNN without FS and the number of iterations.

In terms of these specific evaluation indexes, HHO-FKNN without the FS model has the best results and the smallest variance, followed by GWO-FKNN with FS and HHO-FKNN with FS are the same. Their values are both 0.83 percentage points lower than HHO-FKNN without FS. The next models are RF, SVM, and KELM. The worst is GWO-FKNN without FS, which is only 90.00%. GWO-FKNN without FS has the most considerable variance, reaching 0.2108. As per the results observed in Figure 4, we observe that the HHO-FKNN with the FS model can speedily and unceasingly jump out of local minima, which leads to better accuracy rates. This also indicates the HHO-FKNN with the FS method makes a delicate balance between exploratory and exploitative leanings.

The new framework takes advantage of the dynamic and time-varying operations of HHO when shifting from exploratory drifts to intensification tendencies. Compared to other methods such as GWO, there is no highly focused intensification in HHO, which may result in immature convergence. Generally speaking, when HHO can smoothly shift from its initial exploration to the four stages of its exploitation, it shows a high local optima avoidance rate. Besides, there is a levy flight pattern within HHO’s search phases, which helps this method scan the explored parameters’ vicinity accurately. Then, after determining potential weights, it can enhance the quality of them using its greedy operation. Due to these reasons, we observed a very satisfactory performance within the parameter optimization and feature selection phases. By further observing the curve in Figure 4, it was found that the HHO-FKNN without the FS model is liable to fall into a local optimum, and the accuracy is not as high as that of HHO-FKNN with FS. Although the GWO-FKNN with the FS model ranks second in accuracy, it tends to fall into a local optimum in the late search period. Among all algorithms, GWO-FKNN without FS has the lowest accuracy that is much smaller than that of the GWO-FKNN with the FS model. With continuous iteration, its accuracy improvement is not apparent, and it is easy to fall into a local optimum.

## IV. Discussions

HHO-FKNN machine learning model has been established for clear discrimination between severe and non-severe patients and identified several critical indicators, including advanced age, Hypertension, acute liver injury, and CD4 T cells, CD8 T cells, and fatigue. The results were consistent with previous research. Furthermore, HHO-FKNN can help determine the severity of the COVID-19 patients’ condition quickly and help inform clinical decision-making.

Research studies conducted based on SARS and Middle East Respiratory Syndrome (MERS) infection identified advanced age as a critical factor in determining recovery [121]–[123]. It has been reported that compared with younger macaques inoculated with SARS-CoV, older macaques showed a more robust host antiviral response, which is positively related to inflammation-related genes and type I interferon [124]. Additionally, accumulating evidence shows that the incidence of severe infection and severe sepsis increased with advanced age [125], [126]. It has been generally believed that age-dependent defects in T- and B-lymphocyte function could contribute to increased risk of infection, and over-secretion of type 2 cytokines may stimulate enhanced viral replication [127]–[129]. Taken together, advanced age has adverse impacts on disease recovery.

We are also alarmed about whether patients with cardiovascular illness are at bigger risk for COVID-19. A large population-based study from Canada reported that more than 600 Middle East Respiratory Syndrome Coronavirus (MERS-CoV) cases suggested that hypertension and diabetes mellitus are prevalent in about 50% of the severe cases [130]. Hypertension was an independent predictor of many diseases, including acute coronary syndrome, acute ischaemic stroke, and recurrent colorectal adenoma [131]–[133]. However, the literature on the effects of Hypertension on SARS, MERS, and COVID-19 is scarce. With the spread of COVID-19 and an increase in the number of cases, mortality was higher among COVID-19 infected individuals with comorbidities such as Hypertension, diabetes mellitus, coronary heart disease, cerebral infarction, and chronic lung disease [134]. For example, Chen and colleagues found that in 99 COVID-19 cases, forty percent of patients had cardiovascular and cerebrovascular diseases [135]. Huang *et al.* reported that 20% of COVID-19 patients have diabetes mellitus [134]. In line with these findings, Li *et al.* also revealed that the proportion of COVID-19 patients with Hypertension was two-fold higher in severe cases compared to non-severe cases [136]. Thus, these findings demonstrate that chronic cardiovascular comorbidities with COVID-19 patients were more likely to have a poor prognosis.

A high incidence of acute liver injury (ALI) in SARS patients has been reported (up to 60%). Also, it has been reported in patients infected with MERS-CoV [137], [138]. Several clinical observations have suggested that liver impairment commonly occurs in patients with COVID-19 [135], [139], [140]. These data show that hepatic comorbidities complicated 2–11 % of COVID-19 patients. Elevated serum levels of alanine aminotransferase (ALT) and aspartate aminotransferase (AST) have been reported in 14–53% of COVID-19 cases. ALT and AST are the most frequently used parameters of liver function. A study with many patients from multiple centers in China suggested that compared to the non-severe patients, ALT and AST levels were significantly increased in the severe patients [141]. In a study of intensive care unit (ICU) patients, it was shown that AST levels increased significantly in patients with ICU compared with non-severe patients [2]. Huang and colleagues revealed that the pathological Liver profile of COVID-19 patients, including moderate microvascular steatosis and mild lobular and portal activity, might have been attributable to SARS-CoV-2 infection. These findings demonstrate a relationship between liver impairment and COVID-19, which plays a critical role in disease progression and is associated with risks of COVID-19 [142].

Lymphocytes are critical immune cells divided into three subsets: T lymphocytes, B lymphocytes, and natural killer (NK) cells [143]. Lymphocytes are a kind of white blood cell that plays a pivotal role in adaptive immune function and is a central component of the immune system [144]. The immune response can recognize and remember antigens to eliminate invading bacteria, viruses and so on. Zhou *et al.* reported that CD4 T cells could reflect the body’s immune function. The lower level of the CD4 T cells indicates that the patient immune function level is low [145]. COVID-19 and SARS-CoV belong to coronavirus, and the genomic sequence similarity between COVID-19 and SARS-CoV is very high [146]. For instance, He and colleagues demonstrated that the CD4 T cells, CD8 T cells in peripheral blood were decreased significantly in SARS coronavirus-infected individuals lymphocyte counts may aid with predicting the severity and clinical outcomes [147]. Therefore, similar to SARS coronaviruses, we speculate that COVID-19 may also destroy the immune system, resulting in T lymphocyte immune deficiency. However, the mechanism of the reduction of peripheral blood lymphocytes in patients with COVID-19 is not precise.

However, this research’s findings should be interpreted with thoughtfulness and carefulness due to several potential limitations that we faced during this research. First, this research’s sample size is limited; thus, further research is required to improve diagnostic accuracy. It is scheduled that the HHO-FKNN can be further improved and optimized using a large number of patients from multiple centers. Second, in this study, we focused our investigation only on severe and non-severe patients. Future studies need to focus on discriminating more types of COIVD-19. Third, the features of COIVD-19 involved, though, are likely to be limited. We recommend the inclusion of more metrics of COIVD-19 status such as blood routine, blood biochemistry, arterial blood gas analysis, coagulation function, and systemic inflammatory markers in future studies.

## V. Conclusion and Future Works

Based on patients’ necessary information, pre-existing diseases, symptom, immune index, and complication, this study established a useful HHO-FKNN model to distinguish the severity of COVID-19, of which innovations are as follows: on the one hand, it is proposed for the first time to use the immune index to distinguish the severity of COVID-19, and on the other hand, the HHO algorithm is used for the first time to screen the parameters and features of the FKNN simultaneously. According to the experimental results, the proposed method shows higher prediction accuracy and more stable performance than other machine learning algorithms on the COVID-19 severity prediction problem to select the key factors with more discriminating ability simultaneously. For future work, we will first try to apply the proposed HHO-FKNN to the COVID-19 pre-diagnosis problem and then try to apply it to solve other infectious disease prediction problems.

For future work, the proposed HHO can be wrapped with other popular learning methods such as extreme learning machines [72], [73], [90], [148]–[150], support vector machines [151]–[155], and convolutional neural networks [95], [156]–[158] for the COVID-19 diagnosis task. Furthermore, the improved version of HHO can be developed on some other real fields like video coding optimization [159], feature information fusion [160], social evolution modeling [161], recommender system [162], text clustering [163], unsupervised band selection [164], which are also exciting topics that worthy of the investigation soon.
